# Association of angiotensin-converting enzyme gene I/D polymorphism with steroid responsiveness in childhood nephrotic syndrome

**DOI:** 10.4103/0971-4065.75215

**Published:** 2011

**Authors:** P. Prasun, N. Prasad, G. Tripathi, T. Jafar, S. Sharda, S. Gulati, S. Agrawal

**Affiliations:** Department of Medical Genetics, Sanjay Gandhi Post Graduate Institute of Medical Sciences, Lucknow, Uttar Pradesh, India; 1 Department of Nephrology, Sanjay Gandhi Post Graduate Institute of Medical Sciences, Lucknow, Uttar Pradesh, India

**Keywords:** Nephrotic syndrome, ACE gene polymorphisms, steroid resistance

## Abstract

The aim of the study was to study the distribution of angiotensin-converting enzyme (ACE) gene insertion/deletion (I/D) polymorphism, and its association with steroid responsiveness in children with idiopathic nephrotic syndrome (INS). One hundred twenty-five children with INS were classified into two groups: steroid-sensitive nephrotic syndrome (SSNS: *n* = 90) and steroid-resistant nephrotic syndrome (SRNS: *n*=35). The control group consisted of 150 unrelated healthy children. Genomic DNA was extracted from peripheral leucocytes by the standard salting-out method. ACE genotyping was performed and ACE genotypes DD, ID, and II were compared between different groups. The frequency distribution of the DD genotype was significantly increased in children with INS compared to control subjects (*P* = 0.0012) while the difference was not significant (*P* = 0.071) between SSNS and control subjects. The frequency distribution of the DD genotype was significantly high in the SRNS group compared to control subjects (*P* < 0.0001). The distribution of the DD genotype was high in SRNS compared to SSNS group patients (*P* = 0.016). In conclusion, the presence of the DD genotype may predict risk for steroid resistance in childhood INS.

## Introduction

Childhood idiopathic nephrotic syndrome (INS) patients are broadly categorized into two groups: steroid-sensitive nephrotic syndrome (SSNS) and steroid-resistant nephrotic syndrome (SRNS). Steroid responsiveness is the most important prognostic indicator of INS. The SSNS patients carry good prognosis while SRNS patients tend to progress to renal dysfxnction and end stage renal disease. Angiotensin II may contribute to the progression through its action to increase systemic and glomerular blood pressure, proliferation and matrix production by renal cells, tubulointerstitial fibrosis, and glomerulosclerosis.[1,2] A number of mechanisms have been shown to underlie the pathogenesis of angiotensin II effects on the transglomerular passage of protein. These include modulation of the efferent arteriolar tone, intraglomerular pressure, and glomerular plasma flow as well as changes in the ultrafiltration coefficient and size-dependent barrier functions.[3,4] The functional receptors of angiotensin II have been demonstrated on glomerular podocytes[5] and angiotensin II may directly contribute to the pathogenesis of proteinuria through increased expression of the podocyte slit pore protein, nephrin.[6] The administration of angiotensin II induces proteinuria in rats[7] and angiotensin-converting enzyme inhibitors (ACEI) and angiotensin receptor blockers (ARB) reduce proteinuria in patients with INS.[8] The reduction of proteinuria with ACEI and ARB in patients with proteinuria stresses the role of renin–angiotensin system (RAS) in the pathogenesis of these syndromes.

The levels of angiotensin II depend upon ACE levels. The ACE gene has insertion/deletion (I/D) polymorphism, defined by the presence or absence of the 287-base-pair *Alu* repetitive sequence in intron 16. The ACE levels are highest in individuals with the DD genotype and lowest with the II genotype.[9] It has been shown that the DD genotype is associated with progressive renal dysfunction in diabetic nephropathy and IgA nephropathy.[10,11] There are few studies evaluating the relationship between steroid responsiveness and ACE gene polymorphism in INS in children.[12–14] Therefore, we studied the distribution of the ACE I/D genotype in children with INS and healthy controls and its association with steroid responsiveness in these patients.

## Patients and Methods

The study was conducted at Department of Nephrology and Medical Genetics of Sanjay Gandhi Postgraduate Institute of Medical Sciences, Lucknow, India. One hundred twenty-five children (age range 1–16 years) of NS and 150 unrelated normal healthy children from north India were included for the study. An informed written consent was obtained from both patients and controls. If the children were less than 15 years of age, consent was obtained from the parents or guardians. The study was approved by the institute ethics committee.

### Definitions

Nephrotic syndrome was defined as per the criteria laid by International Study of Kidney Disease in Children.[15] The NS in children was defined as proteinuria of 40 mg/m^2^/h or a spot urine protein (mg)/ creatinine (mg) ratio of 2 in first morning urine sample. Remission was defined as the urinary protein excretion of <4 mg/m^2^/h or urine dipstix nil/trace for three consecutive days. Relapse was defined as the urinary protein excretion of >40 mg/m^2^/h or urine dipstix 2+ or more for three consecutive days. Frequent relapses (FR) were defined as two or more relapses within 6 months of initial response or four or more relapses within any 12-month period. Steroid dependence (SD) was defined as two consecutive relapses occurring during the period of steroid tapering or within 14 days of its cessation.

All children were treated with daily dosages of prednisone 60 mg/m^2^ of the body surface area for 6 weeks, followed by 40 mg/m^2^ given on alternate days for 6 weeks and finally by various steps of tapering-off on alternate days. Relapses were treated with prednisone 60 mg/m^2^/day until remission, followed by 40 mg/m^2^on alternate days. In steroid-dependent patients, maintenance alternate day prednisone was given. The alternate dose was gradually tapered off to determine each patient’s individual threshold at which relapse occurred. Steroid resistance was defined as no response to therapy after 8 weeks of a high dose (60 mg/m^2^) of prednisolone therapy. Kidney biopsy was performed in the steroid-resistant cases after obtaining consent. Genotyping was performed in all nephrotic patients and controls.

Genomic DNA was extracted from peripheral leucocytes by the standard salting-out method.[16] In order to determine the ACE genotype, a genomic DNA fragment on intron 16 of the ACE gene was amplified by polymerase chain reaction. The sequences of the sense and antisense primers were 5- CTGGAGACCACTCCCATCCTTTCT-3’ and5’-GATGTGGCCATCACATTCGTCAGAT-3’, respectively. Fragments without insertion (D allele) and with insertion (I allele) of 190 and 490 base pairs, respectively, were detected on the 2% agarose gel containing ethidium bromide.

All continuous data were expressed as mean±SD. Genotype comparisons of different groups were made using the chi-square test. A *P*-value of <0.05 was considered to be significant. The statistical analyses were performed by using software *EpiInfo* version 3.3.2.

## Results

Out of 125 children with INS, 90 children had SSNS (73 boys, age 5.3±4 years at the onset of NS) and 35 had SRNS (24 boys, age 10.9±3.8 years at the onset). Of the 90 SSNS patients, 47 had infrequent relapses (IFR), 20 FR, and 23 had SD. Out of 35 SRNS cases, a kidney biopsy was available in 29 [21 FSGS, and 8 MCD] and 6 children were not biopsied because parents had not consented for kidney biopsy.

The DD, ID, and II genotypes were found in 11 (7.3%), 39 (26%), and 100 (66.7%) of the 150 healthy controls, and in 27 (21.6%), 48 (38.4%), and 50 (40%) of the 125 INS group patients, respectively. The DD, ID, and II genotypes were observed in 14 (15.5%), 33 (36.7%), and 43 (47.8%) of the 90 SSNS group patients; and 13 (37.1%), 15 (42.9%), and 7 (20%) of the 35 SRNS group patients, respectively. The NS patients as a group had significantly higher percentage of the DD genotype (*P*=0.0012) than the controls [Table 1]. Similarly, SRNS patients had a significantly higher percentage of the DD genotype (*P*<0.0001) than the control group. The frequency distribution of the DD genotype was also significantly high in SRNS compared to SSNS group patients (*P*=0.016). However, there was no significant difference in the distribution of the DD genotype between the SSNS and the control group (*P*=0.071). The frequency distributions of different genotypes among the SSNS subgroups [IFR (*n*=47), FR (*n*=20), SD (*n*=23)] were not different. The frequency distribution of genotype DD, ID, and II in 21 patients with FSGS was 4 (19%), 9 (44%), and 8 (37%), respectively, while in 8 patients with MCD, it was 1 (6%), 3 (38%), and 4 (56%), respectively (*P*=0.42).

**Table 1:** Distribution of ACE genotypes in steroidsensitive and steroid-resistant children with idiopathic nephrotic syndrome, and control groups

Polymorphisms Control (%)		Nephrotic syndrome (%)		
	(n=150)	Total (n=125)	SSNS (n=90)	SSNS (n=35)
DD	11 (7.3)	27 (21.6)^a^	14 (15.6)^b^	13 (37.1)^c^,^d^
II/ID	139 (92.7)	98 (78.4)	76 (84.4)	22 (62.9)

a *P* = 0.0012, total patient versus control

b *P* = 0.071 SSNS versus control;

c *P* < 0.0001 SRNS versus control;

d *P* = 0.016 SRNS versus SSNS;

I = Insertion; D = Deletion, SSNS = Steroid-sensitive nephrotic syndrome; SRNS = Steroid-resistant nephrotic syndrome

## Discussion

In this study, the frequency distribution of the DD genotype was high in nephrotic patients compared to controls. It was also observed that the DD genotype was significantly high in SRNS patients compared to SSNS patients. The SSNS and SRNS patients behave differently and the progression to renal functional impairment and end-stage renal disease is more common in SRNS patients compared to SSNS patients. Genetic factors are supposed to affect many aspects of INS, i.e., susceptibility for acquiring disease, treatment responses, histological findings, and disease progression. There is significant evidence that the RAS system is involved in the pathogenesis of progressive renal diseases.[17,18] An increased angiotensin II level has harmful effects on renal hemodynamics and contributes to glomerulosclerosis. It has been observed that angiotensin enhances proteinuria, and the use of ACE inhibitors has been demonstrated to reduce nephrotic or nonnephrotic proteinuria.[7,19] The presence of the D allele is associated with increased circulating angiotensin levels.[20] There are studies demonstrating a link between the D allele and susceptibility to some primary glomerulopathies. It has been shown that the DD genotype is associated with a more severe renal course.[21,22] The II genotype has been reported to be associated with more favorable prognosis and decreased proteinuria in patients with IgA nephropathy.[23]

There is limited information on ACE gene I/D polymorphism in childhood INS.[12–14] Moreover, the results are contrasting and a consensus is yet to be drawn. Lee *et al*.[12] reported that the distribution of the ACE genotype in patients with MCD was not different from that of the control group. However, the DD genotype was more frequent in patients with FSGS than in patients with MCD. It was concluded that the DD genotype might be a risk factor for the poor response to steroid therapy and the development of chronic renal failure.[12] We have also observed a higher DD genotype frequency in FSGS patients compared to MCD patients. However, the difference was not significant because of less number of patients. Oktem *et al*. reported that there was no difference in the ACE genotype between children with FSGS and normal controls. But, the frequency of the DD genotype was higher in FSGS patients with a declining renal function than in those with a stable renal function.[13] Serdaroglu *et al*. have shown the frequency of the D allele to be higher in INS patients than healthy controls, and the DD and ID genotypes were related to frequent relapses. However, ACE I/D polymorphism was not found to be important in laboratory and histological findings and progression of the disease in children with INS.[14]

The higher frequency of the DD genotype in patients with INS patients compared to healthy controls; in SRNS group compared to controls; but no significant difference between SSNS patients and control group suggest the fact that the higher incidence of the DD genotype in total patient group may be solely attributed to the SRNS group of patients. The DD genotype was associated with poor responsiveness to steroids. A similar observation, in terms of steroid responsiveness, was made in patients with FSGS by Frishberg *et al*.[22] They observed remission in two-thirds of children with II genotype compared to only 24% of those with ID or DD genotype in their study. The most frequent histological finding in SRNS was FSGS. The MCD and FSGS are thought to be different stages of the same disease. The increased RAS activity may lead to the progression of MCD to FSGS as well as steroid resistance. However, the ACE DD genotype is associated with increased ACE levels which are generally twice as high as those found in the II genotoype and had an intermediate level in the ID genotype. Angiotensin II, the most active product of the RAAS, may contribute to disease progression through local and systemic hemodynamic regulation, renal mesangial cell growth, extracellular matrix synthesis and degradation, and inflammatory processes. These effects are mediated by release of several factors including TGF-beta, plasminogen activator inhibitor-1, and monocyte chemoattractant protein-1 and activation of various nuclear transcription factors including activator protein 1 and nuclear factor kB. The exact mechanism of steroid resistance in the DD genotype is not known. It is possible that during the course of disease progression, DD genotype patients develop steroid resistance. The mode of action of steroid in INS was unclear until recently. The functional receptors of angiotensin II have been demonstrated on glomerular podocytes[5] and angiotensin II may directly contribute to the pathogenesis of proteinuria through modulating the expression of the podocyte slit pore protein, nephrin.[6] Fujji *et al*.[24] have demonstrated defective nephrin transport following endoplasmic reticulum (ER) stress in podocyte causing the alteration of nephrin N glycosylation, which may be the underlying pathogenesis of proteinuria in INS. Dexamethasone may restore this imbalance by stimulating the expression of mitochondrial genes, resulting in the production of ATP which is an essential factor for proper folding machinenary aided by the ER chaperones. It is possible that increased angiotensin II in the DD genotype may be causing steroid resistance through increased production of nephrin and nephrin N glycosylation at the podocyte.

In conclusion, our results indicate that genotype DD is associated with steroid resistance and the presence of the DD genotype in childhood NS patients may serve as a predictive risk factor for steroid resistance and thus a poor clinical response.
